# The clinical significance of allergen-specific IgG4 in allergic diseases

**DOI:** 10.3389/fimmu.2022.1032909

**Published:** 2022-10-25

**Authors:** Lu Qin, Lan-Fang Tang, Lei Cheng, Hui-Ying Wang

**Affiliations:** ^1^ Department of Pulmonology, the Children’s Hospital of Zhejiang University School of Medicine, Hangzhou, China; ^2^ Department of Otorhinolaryngology & Clinical Allergy Center, The First Affiliated Hospital, Nanjing Medical University, Nanjing, China; ^3^ Department of Allergy and Clinical Immunology, the Second Affiliated Hospital of Zhejiang University School of Medicine, Hangzhou, China

**Keywords:** IgG4, Fab-arm exchange, allergen specific immunotherapy, food allergy, eosinophilic esophagitis

## Abstract

IgG4 is a subclass of IgG antibody with a unique molecular feature of (Fragment antigen- binding) Fab-arm exchange, allowing *bi*specific antigen binding in a *mono*-valent manner. With low binding affinity to C1q and Fcγreceptors, IgG4 is incapable of forming immune complexes and activating the complement pathway, exhibiting a non-inflammatory feature. IgG4 is produced similarly to IgE and is considered *a* modified reaction to IgE class-switching response under certain conditions. It could also counteract IgE-activated inflammation. However, the clinical significance of IgG4 in allergic diseases is complex and controversial. Three viewpoints have been suggested to describe the role of IgG4. IgG4 can act as a tolerance–inducer to play a protective role under repeated and rapid incremental dosing of allergen exposure in allergen immunotherapy (AIT), supported by allergies in cat raisers and venom desensitization in beekeepers. Another viewpoint accepted by mainstream specialists and guidelines of Food Allergy and Management in different countries points out that food-specific IgG4 is a bystander in food allergy and should not be used as a diagnostic tool in clinical work. However, eosinophilic esophagitis (EoE) investigation revealed a direct clinical relevance between physiopathology and serum IgG4 in cow milk and wheat. These factors indicate that allergen-specific IgG4 plays a multifaceted role in allergic diseases that is protective or pathogenic depending on different allergens or exposure conditions.

## Introduction

Subclasses of IgG antibodies were discovered in the 1960s (1). IgG4 is one of the subclasses detected recently and constitutes about 5% of total IgG, the smallest portion among all IgGs in serum. Although these different subtypes of IgG have more than 90% identical amino acids, they display different immunological effects such as immune complex formation and complement activation. Previous studies have revealed that different IgG subclasses are associated with different antigens. IgG1and IgG2 subclass is usually associated with the response to bacterial polysaccharides. IgG3 is a potent pro-inflammatory antibody to induce effector function (2), while IgG4 is for non-microbial allergens.

IgG4 is conventionally considered non-inflammatory or anti-inflammatory because it cannot form large immune complexes and activate complement component pathways (3). Thus, its monoclonal antibodies are applied as therapeutics in tumors, rheumatic arthritis, and asthma (4–10). However, IgG4 is also associated with various diseases, including IgG4-related diseases, autoimmune and hematologic disorders, parasitic infections, and neoplasms (11, 12). A recent study showed that serum IgG4 levels could predict COVID-19 related-mortality (13). These studies indicate that IgG4 is also pathogenic and can induce Th2 inflammation following final fibrosis.

The clinical significance of IgG4 in allergic diseases is controversial. The earliest recognition of IgG4 in allergies was from Allergen-specific immunotherapy (AIT) data, demonstrating a tolerance-inducing function (14–16). However, later clinical observations revealed that allergen-specific IgG4 was associated with allergic sensitization and correlated to allergic diseases, such as allergic rhinitis (AR), asthma, atopic dermatitis (AD), and anaphylaxis (17–23). IgG4 levels may vary significantly in healthy individuals (24), strongly hindering its clinical application as a diagnostic tool. Nevertheless, recent findings from molecular structure and clinical investigations have brought new insights into this subset of IgG antibodies in allergic diseases.

The present study provides a comprehensive review of recent studies on IgG4, its structure features, immune biology, interaction with IgE, and different roles in different allergic statuses.

## Unique molecular feature of Fab-arm exchange and impact on its immune response

**Figure 1:**
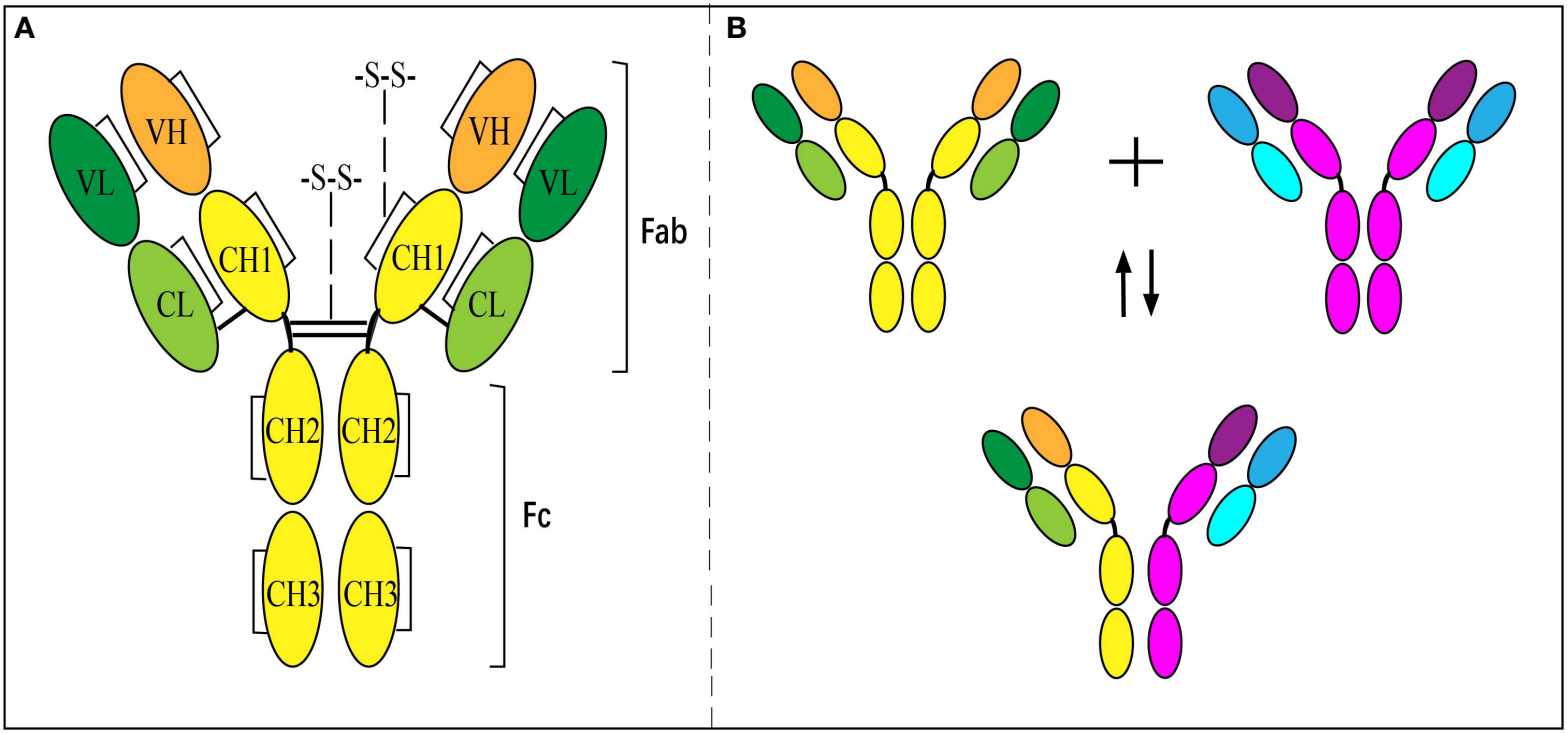
Structure of IgG4 antibody and Fab-arm exchange. **(A)** Inter- and intra-chain disulfide bonds give structure to IgG4. **(B)** Antibodies separate into ‘half-molecules’, each comprising one heavy and one light chain. Half-molecules recombine to form bi-specific antibodies.

IgG4 has a unique molecular feature distinguished from other subclasses of IgGs, which results in diversity in the immune response. Like other subclasses of IgG, the basic molecular structure of IgG4 is composed of two light chains (-25 kD) and two heavy chains (-50 kD) paired together (**Figure 1A**) (25, 26). The light chain has one constant domain (C_L_) and one variable domain (V_L_), while the heavy chain consists of 3 IgG4-specific constant domains (C_H_, including C_H_1, C_H_2, and C_H_3) and one variable domain (V_H_). The variable domains of the heavy (V_H_) and light chain (V_L_), with the attached C_L_ and C_H_1 domains, respectively, formed the ‘fragment antigen-binding region’ (Fab-region), which is highly specific for one epitope. However, unlike other subclasses of IgG composed of two same chains, IgG4 are dynamic molecules that dissociate into two half molecules with one light chain and one heavy chain, then reassemble with another half one. It is called FAE and results in a bi-specific binding antibody (**Figure 1B**) (27, 28). The phenomenon is due to the serine replacing proline in the hinge region at positions 288 and 331 (29, 30), which makes the disulfide bond unstable. The half-molecular form of IgG4 exists up to 10% in circulation, and the main body is full-molecular IgG4 with bispecific epitopes (31). The pathogenesis and the regulating factors of FAE are still unclear. The exchange could occur *in vivo* or *in vitro* under some conditions or spontaneously (28).

This unique feature of the bi-specificity of IgG4 enables it to combine two different allergens *via* one molecule (3, 28) and behave as a *mono*-valent antibody. AIT using different antigens showed the best demonstration of this (25). *In vitro* experiments have also exhibited that IgG4 is incapable of forming complexes *via* immuno-precipitation (28, 32). Therefore, the complement cascade cannot activate to cause cell lysis and the classical pathway.

The site of P331 is the binding site to C1q, and the amino acid mutation caused the low affinity of IgG4 to C1q and the consequent tendency to interact with other immunoglobulins (33). Thus, IgG4 can prevent the biological effect of the complement IgG1 subclass. Moreover, IgG4 has no affinity to the receptors FcgRIIIa and FcgRIIIb (34, 35). These features render IgG4 an anti-inflammatory or tolerance-genic antibody.

It was noted that FAE produces 2 types of IgG4 since recombination is a random process. One has bi-specific epitopes composed of 2 arms with different allergen sources. Another one might have 2 arms with a similar allergen source, meaning it might behave in a bivalent manner and be pathogenic in some inflammatory diseases, such as allergic diseases.

## IgG4 synthesis and its interaction with IgE

The production of IgG4 seems to have a very similar pathway to IgE. Both are part of the Th2 immune response, triggered by allergen exposure and produced by a class switch on B cells dependent on IL-4/IL-13. Th2 cytokines (e.g., IL-5, IL-6, IL-9, and IL-13) that involve IgE production have similar effects on IgG4 production. However, what predominates the diverging to IgE or IgG4 is still unclear. It was speculated that the allergen stimulus might be the most important factor in initiating the switch to IgE or IgG4. Antibody response to natural cat allergen exposure provides a clear picture, with high IgG4 and low IgE responses in cat raisers. AIT further exhibits a dynamic switch of IgE production to IgG4 production after repeated and rapid increments of allergen exposure. Relevant studies found that IL-10 played a critical role in this shift of IgE to IgG4. *In vitro* experiments showed a culture of peripheral blood monocytes with the addition of IL-10 could achieve IgG4 production over IgE, and IL-10-producing T regulatory cells suppressed IgE production (36, 37). The IgG4 production after AIT is correlated to the significant elevation of serum IL-10. IL-21, a pleiotropic cytokine, is also recently found to have a role in shifting IgE response to IgG4 response (38). Thereby, a modified Th2 response was nominated to describe this phenomenon (39), which helps illustrate the clinical significance of IgG4, as discussed later.

**Figure 2:**
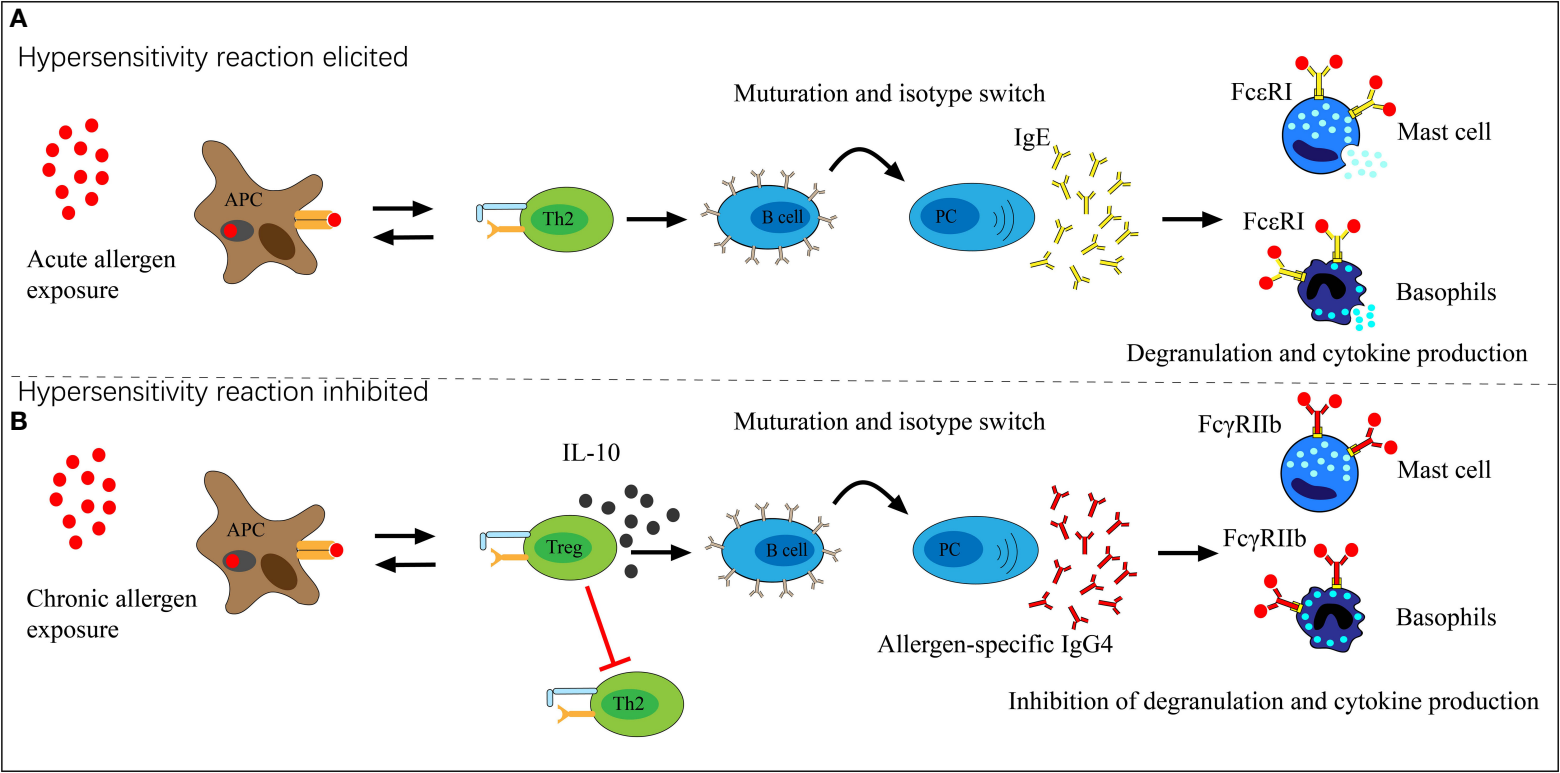
The role of allergen-specific IgG4 in allergen exposure. **(A)** Acute allergen exposure may lead to IgE-mediated histamine-induced hypersensitivity reactions by high-affinity receptor FcϵRI. **(B)** Chronic allergen exposure may induce IgG4 formation and allergen binding by IgG4, thereby binds to the inhibitory receptor FcγRIIb which prevents hypersensitivity reactions and induces allergen tolerance.

The immune biological role of IgG4 appears to counteract IgE through different pathways. First, IgG4 can block IgE binding to specific allergens in serum through competition, though the difference in repertoires may influence the results (40, 41). Studies of food allergens showed that IgE and IgG4 had similar binding patterns in peanut, milk, egg, and lentil allergy (42–45). Moreover, serum IgG4 responses to AIT were well documented as blocking allergen binding to receptor-bound IgE on antigen-presenting and effector cells (46, 47). The second and more critical pathway is the direct inhibition of IgE-mediated mast cell activation (**Figure 2**). IgE binds to the high-affinity receptor FcϵRI and triggers the degranulation reaction of mast cells. In contrast, IgG4 binds to the inhibitory receptor FcγRIIb to inhibit the above effects at both the initiation and effector phases of allergic immune responses (48–50). A grass pollen-specific IgG4 antibody isolated from a patient undertaking AIT blocked the interaction between allergen and IgE and inhibited basophil activation. In addition, it inhibited the IgE-facilitated antigen presentation by B cells which promotes allergic inflammation and the engagement of membrane CD_23_ by IgE-allergen complexes (46). Furthermore, the unique capacity of IgG4 for FAE, which means dissociation and re-pairing of IgG4 Fab arms, creates bispecific antibodies capable of interfering with IgE-allergen complex formation (28, 51). This interaction with IgE may contribute to the harmless or protective appearance of IgG4 in allergic diseases, but the clinical implication is likely much more complex.

## Development of IgG4 and its association with allergic diseases

IgG4 could be detected in the serum of healthy subjects with a variance of a wide range. Multiple birth cohort studies have discovered the expression of IgG4 in the serum of infancy at a low level, rising to a peak at 2-3 years of age and declining after that (20, 52–55). However, higher levels or the ratio to total IgG of allergen-specific IgG4 have been associated with atopy in children. Despite the decline of the total IgG and IgG4 in egg and milk, the ratio of IgG4 to total IgG goes up from 15% at six months to 50% at 5 years in children with one parent of atopy (56). In children sensitized to foods, specific IgG4 peaks earlier and persists for 8 years and longer (20). Furthermore, maternal atopy is linked to IgG subclass antibodies to food and inhalant allergens in cord blood (52).. The studies showed that the maternal food-specific IgG4 antibody titer was related to allergy in children with AD (53). These data indicated that IgG of all subclasses, especially IgG4, is elevated earlier in children of atopic mothers, and the atopy in children is associated with elevated maternal IgG4 antibodies. However, the IgG4 response to different allergens is varied. IgG4 for food allergens was higher than inhalant allergens (e.g., pollen) (57). Among the food allergens, the level of IgG4 in cow’s milk was dramatically higher than in peanuts (58).

This association between IgG4 and allergy brings vital interest in its clinical significance, which is complex and controversial. Accumulated data from clinical observations demonstrate three viewpoints on the correlations of IgG4 in different statuses (**Table 1**). The first viewpoint is that IgG4 is protective and tolerogenic. Another perspective is that IgG4 has no value in clinical diagnosis and therapy as a bystander. However, recent studies found that IgG4 may play a pathogenic role in many allergic diseases, including respiratory, skin, and food allergies, as discussed in more detail below.

**Table 1:** Three viewpoints of clinical correlation of IgG4 in allergic diseases.

Three viewpoints	Related studies	References
Protective or tolerancegenic	Studies of allergen immunotherapy (AIT)	Ref 59–67.
	Studies of cat raisers and occupational exposure of bee and rodent allergens	Ref 39, 68–71.
	High IgG4/IgE correlated tolerance	Ref 14–16.
Bystander: No value	Guidelines of food allergy and management	Ref 72, 73.
	Level of IgG4 is not related to clinical symptoms	Ref 17, 18, 74–81.
Pathogenic	Higher IgG4 correlates to clinical symptoms	Ref 23, 84.
	Studies of chronic rhinosinusitis eosinophilic (CRS)	Ref 83, 86.
	Studies of eosinophilic esophagitis (EoE)	Ref 91–103.

### The protective role of IgG4 in allergic diseases

Anti-inflammation and tolerance-inducing in allergic diseases are the most common recognitions of IgG4, mainly based on its IgE-counteracting effector role and clinical observation of AIT. Just after the discovery of IgG4 in the early 1970s, three independent groups reported a significant increase in IgG4 levels after AIT (59, 60). In the following four decades, more data on AIT has accumulated a convincing body of evidence for the recognition of IgG4 as a biomarker of the tolerance inducer in AIT (61). These clinical investigations of AIT demonstrated a comprehensive overview of IgG4’s protective function in different types of allergens, including food allergens such as milk and eggs (62–65), inhalant allergens such as pollen or mite extracts (14), as well as venom proteins (66). These AIT studies demonstrated modified Th2 response under high concentrations of allergen exposure.

Anecdotal stories from people who raise cats as well as those of beekeepers also strongly support evidence of immune response to natural exposure. Hesselmar et al. reported a reduced risk of cat allergy in cat raisers (67), confirmed by Platts-Mills and Renand (39, 68). Plattes-Mills et al. further comprehensively studied the exposure concentration-related response of IgG4 with low sensitization of cat allergens. Beekeepers have demonstrated occupational exposure and natural desensitization with increased specific IgG4 (69), similar to tolerant animal laboratory workers chronically exposed to rodent allergens (70, 71).

In addition, the primary references of IgG4 showed that the appearance of IgG4 antibodies is usually associated with a decrease in symptoms. In a study on the mechanism of milk allergy and tolerance, it was reported that atopic individuals with milk protein tolerance were accompanied by increased food-specific IgG4 antibodies and a decrease in corresponding IgE (14). Children sensitized with aero-allergen-specific IgE antibodies also reported having no symptoms under a higher IgG/IgE ratio in sera, which inhibits the activation of basophils sensitized with aero-allergen IgE (15). Santos and colleagues found that higher levels of peanut-specific IgG4 correlate with tolerance in those subjects with positive specific IgE (16).

### No value in the diagnosis and management of the allergic disease

Most specialists do not accept the clinical value of food allergen-specific IgG4 in food allergy, and its clinical application is vehemently opposed. Almost all guidelines on food allergy outline a negative opinion. A position paper by the EAACI task force from 2008 declared that food-specific IgG4 was not recommended as a diagnostic tool for food allergy (72). The American Academy of Allergy and Immunology Position Statement (AAAI) also holds the same opinion and concluded that using specific and nonspecific IgG4 does not serve as diagnostic and prognostic tests for clinical allergy (73). Therefore, the serological tests for food-specific IgG4 are disputable and seem to not be an indicative diagnostic tool for food allergy.

Studies have revealed the irrelevance of food-specific IgG4 to clinical symptoms of food allergy (74). The testing of food-specific IgG4 failed to distinguish double-blind placebo-controlled food challenge (DBPCFC)-positive from negative patients (75, 76). After eliminating IgG-positive foods, the success rates of hypersensitivity reactions like asthma, headache, fatigue, and serous otitis correspond to the high placebo effect of each manipulation in the diet (77). Lichtenstein et al. (78) also showed that IgG-specific mAb were binding to IgG-IgE complexes attached to basophil through IgE bound to the IgE receptor, which showed passive sensitization to anti-IgG could be blocked by previous exposure of the basophils to IgE. These cells cross-link the IgE receptors through the above complexes, which supports the idea that food-specific IgG4 antibody responses do not help assess allergic disease or plan food-elimination diets. A follow-up study on 2-5 year old of children with food allergies demonstrated that the occurrence of serum IgE and IgG antibodies to some food was parallel in most cases (79). Another study reported that none of the patients with positive food-specific IgG4 showed adverse reactions, neither immediate nor delayed, which means food-specific IgG4 does not indicate a food allergy or intolerance (80). High IgA, IgG1, or IgG4 in atopic children was not always consistent with allergic sensitization and atopic diseases (17, 18). Moreover, Calkhoven PG et al. (81) and Eysink PE et al. (82) both describe that food-specific IgG4 is commonly found in children without any clinical manifestation of allergy-like diseases. The outcome of those studies queries the usefulness of testing for food-specific IgG4 and even objects to it as a diagnostic tool in allergic disorders. The main explanation for this bystander effect is that food-specific IgG4 is a natural product of antigen exposure as part of the normal immune response to foods. Increasing evidence disagrees with this viewpoint.

### Pathogenic role of IgG4 in allergic diseases

With the development of techniques in measurement and structural investigations, new features of pathogenic roles of IgG4 in allergic diseases are gradually recognized. Allergies are chronic inflammatory disorders involving various immune cells such as mast cells, eosinophils, and T lymphocytes (83). IgG4 was also found to be higher in patients with AR, asthma, and AD than in non-allergic subjects and had a positive correlation to allergic diseases (23). Elevation of serum IgG4 has additionally been seen in patients with aspirin-exacerbated respiratory disease (AERD), nasal polyposis, eosinophilia, and celiac diseases (84). Oka et al. conducted a series of studies of clinical investigations and revealed that serum IgG4 level is associated with the severity of chronic rhinosinusitis eosinophilic (CRS), and it could be a novel biomarker to predict post-operation reoccurrence (85, 86). The latest study using microarray immunoassay to assay the binding of IgG4 to lenti1 epitopes found that the signal intensities of positive epitopes were significantly greater in reactive patients than tolerant ones (45). Several studies confirmed that food-specific IgG4 served as an anaphylactic antibody against some foods and inhaled allergens in asthma and other atopic diseases (22, 87, 88). Further research has also demonstrated that some relevant allergic symptoms finally improved or disappeared when foods with positive IgG4 test results were avoided (89, 90).

The most solid evidence of IgG4’s pathogenicity in allergy are studies of Eosinophilic esophagitis (EoE) conducted by the Platts-Mills TAE group in children (91) and Clayton’s group in adults (92). EoE is a chronic Th2 cell-mediated disease of the esophagus that contributes to poor quality of life, dysphagia, and food impactions in all ages, characterized by esophageal dysfunction, and a productive eosinophil inflammation of the esophageal mucosa (91–95). It was first recognized as a form of food allergy by Kelly et al. (96) in 1995 and is now expected as the third form of food allergy suggested by Platts-Mills TAE in Virginia (91). EoE has been well demonstrated as the adaptive Th2-type response to food antigens, with several characteristic cytokines and chemokines in food-specific responses (97–99). In 2014, Clayton et al. (92) reported that high serum IgG4 reacted with milk, wheat, egg, and nuts in adult EOE patients and, more critically. Biopsy specimens demonstrated the deposition of IgG4 in esophageal tissues, which indicated that, in adults, EoE is associated with food-specific IgG4 antibodies. A later case-control study of 20 adult EoE subjects and 10 non-EoE controls was performed and verified this association (100). In addition, another research also confirmed that food-specific IgG4 antibodies are easily collected along the esophageal lumen of EoE patients (101).

The results of serum IgG4 antibodies on EoE pediatric cases were also intriguing. Thomas’ group found both a high prevalence and very high titers of IgG4 antibodies to the cow’s milk proteins alpha-lactalbumin (Bos d 4), beta-lactoglobulin (Bos d 5), and casein (Bos d 8) as well as to gluten from wheat (102). The most persuasive evidence comes from the food avoidance and re-induce test. The symptoms and histopathology of IgG4 deposition disappeared completely after simple avoidance of cow milk and reoccurred after the food was re-introduced. Moreover, successful OIT for food allergies to peanuts, milk, or egg can cause an EoE-like condition with the elevation of food-specific IgG4 antibodies in serum (103). A different group from San Paulo, Brazil, presented the same findings at the American Academy of Allergy, Asthma, and Immunology (AAAAI) meeting in 2019 (104).

The underlying mechanism is still unclear. Factors such as food particles, and the undiscovered pathway of IgG4 to form an immune complex and activate mast cells, might be possible reasons (105, 106).

## Conclusion

IgG4 has multiple profiles in allergic diseases due to its unique molecular feature and consequent biology. The FAE results in a dynamic antibody with *bi-*specific binding capability in a monovalent manner. A similar condition of IgG4 production to IgE induces the switch of IgE/IgG4. IgG4 plays different roles under different clinical conditions. It works as a tolerance inducer in AIT, cat raisers, and beekeepers, a bystander in food allergy, and a pathogenic role in EOE. The underlying mechanism of these multiple faces of IgG4 in allergic diseases is still unclear and needs further investigation. However, it brings new light to the treatment of allergic diseases as a therapeutical target. For EOE, avoidance of food with positive IgG4 is a good strategy to relieve the symptoms. For some allergies, the application of allergen-specific IgG4 antibodies might be an effective approach to creating a blockade.

## Author contributions

LQ drafted the molecular feature, impact, and pathogenic role of the IgG4 section. H-YW drafted all remaining sections. All coauthors reviewed and edited the final manuscript. All authors contributed to the article and approved the submitted version.

## Funding

This study was supported by the National Natural Science Foundation of China (81170016, 81470214 & 82070028), the Zhejiang Provincial Program for the Cultivation of High-Level Innovative Health Talents (2016), and the Innovation Team for the Diagnosis and Treatment of Childhood Asthma.

## Conflict of interest

The authors declare that the research was conducted in the absence of any commercial or financial relationships that could be construed as a potential conflict of interest.

## Publisher’s note

All claims expressed in this article are solely those of the authors and do not necessarily represent those of their affiliated organizations, or those of the publisher, the editors and the reviewers. Any product that may be evaluated in this article, or claim that may be made by its manufacturer, is not guaranteed or endorsed by the publisher.
